# Emergency medical service provider decision-making in out of hospital cardiac arrest: an exploratory study

**DOI:** 10.1186/s12873-017-0136-3

**Published:** 2017-07-25

**Authors:** J. Brandling, K. Kirby, S. Black, S. Voss, J. Benger

**Affiliations:** 1 University of the West of England, Bristol, UK; 2 South Western Ambulance Service NHS Foundation Trust, Dorchester, UK

**Keywords:** Out-of-hospital cardiac arrest, Emergency medical services, Decision making, Resuscitation

## Abstract

**Background:**

There are approximately 60,000 out-of-hospital cardiac arrests (OHCA) in the United Kingdom (UK) each year. Within the UK there are well-established clinical practice guidelines that define when resuscitation should be commenced in OHCA, and when resuscitation should cease. Background literature indicates that decision-making in the commencement and cessation of resuscitation efforts in OHCA is complex, and not comprehensively understood. No relevant research from the UK has been published to date and this research study seeks to explore the influences on UK Emergency Medical Service (EMS) provider decision-making when commencing and ceasing resuscitation attempts in OHCA. The aim of this research to explore the influences on UK Emergency Medical Services provider decision-making when commencing and ceasing resuscitation attempts in OHCA.

**Methods:**

Four focus groups were convened with 16 clinically active EMS providers. Four case vignettes were discussed to explore decision-making within the focus groups. Thematic analysis was used to analyse transcripts.

**Results:**

This research found that there are three stages in the decision-making process when EMS providers consider whether to commence or cease resuscitation attempts in OHCA. These stages are: the call; arrival on scene; the protocol. Influential factors present at each of the three stages can lead to different decisions and variability in practice. These influences are: factual information available to the EMS provider; structural factors such as protocol, guidance and research; cultural beliefs and values; interpersonal factors; risk factors; personal values and beliefs.

**Conclusions:**

An improved understanding of the circumstantial, individual and interpersonal factors that mediate the decision-making process in clinical practice could inform the development of more effective clinical guidelines, education and clinical decision support in OHCA. These changes have the potential to lead to greater consistency.

and EMS provider confidence, with the potential for improved patient outcome from OHCA.

**Electronic supplementary material:**

The online version of this article (doi:10.1186/s12873-017-0136-3) contains supplementary material, which is available to authorized users.

## Background

Approximately 60,000 people suffer an out-of-hospital cardiac arrest (OHCA) in the United Kingdom (UK) each year [1]. There is well-established UK clinical practice guidance, based on the 2015 UK Resuscitation Council Guidelines that indicates when Emergency Medical Service (EMS) providers should commence and cease resuscitation in OHCA [2]. The Joint Royal Colleges Ambulance Liaison Committee (JRCALC) 2016 Guidelines [3] state that advanced life support should be commenced and continued unless any of the following conditions are met:

- The presence of a Do Not Attempt Cardio-Pulmonary Resuscitation Order (DNACPR) or an Advanced Decision stating the patient’s wishes not to undergo attempted resuscitation
- A patient in the final stages of a terminal illness where death is imminent and unavoidable and cardiopulmonary resuscitation would not be successful, but for whom a DNACPR has not been made
- Where ALL the following exist together: 15 min since cardiac arrest, no bystander CPR prior to the arrival of the ambulance, the absence of exclusion factors (drowning, hypothermia, poisoning or overdose, pregnancy), asystole for >30s
- Submersion for longer than 60 min

These guidelines are used by EMS providers to make decisions on whether to commence advanced life support (ALS) and whether to carry on or cease ALS in OHCA. Whilst national guidance for resuscitation exists, variability in resuscitation practice resulting from the inconsistent application of these guidelines is not well understood.

Published clinical outcome data for April 2016 indicate that there is considerable variability in survival to hospital discharge following OHCA between ambulance services. This varies between 4.7% and 16.7% in the English ambulance services [4].

The multifactorial factors that influence EMS decisions to commence and cease resuscitative measures, including provider, patient and situational variables, have been previously commented on, as has the complex nature of decision-making in a continually changing ‘landscape of viability at the extremes of life’ [5].

An examination of literature published to date identified no previous research investigating the views and practice of UK EMS providers in this subject area. However, the influencing factors identified in research from other countries includes: prognostic criteria [6, 7]; OHCA in a public place [7, 8]; perceived futility [8, 9]; personal beliefs [10]; paramedic competence [10]; clinical experience [11, 6] and family and bystanders influence [6–8, 12]. Many of these factors are not considered currently in the UK pre-hospital termination of resuscitation guidelines [3].

A recently published systematic review investigating factors that inform the decision-making of OHCA resuscitation providers when commencing, continuing, withholding and terminating resuscitation in OHCA has noted the absence of the OHCA providers’ perspective in this area [13].

Further study is indicated to understand the influences on UK EMS resuscitation decision makers when commencing and ceasing OHCA resuscitation attempts. We therefore completed a qualitative study to investigate contemporary views regarding decision-making during OHCA by EMS staff in the UK.

### Aim

To explore the influences on UK EMS providers’ decision-making when commencing and ceasing resuscitation attempts in OHCA.

## Method

A qualitative study utilising focus groups and case vignettes was used to examine the decision-making of UK EMS providers (paramedics) in relation to the commencement and cessation of resuscitation attempts in OHCA. South Western Ambulance Service NHS Foundation Trust (SWAST) provided research approval.

Four focus groups were convened with 16 clinically active EMS providers recruited from within SWAST. Adverts were placed in an internal bulletin and EMS providers were recruited from emergency response vehicles and specialist teams as well as clinically active teaching and management staff. The initial three focus groups were formulated to test and refine the research methodology and consisted of non-standard EMS providers (i.e. those in management, teaching or specialist EMS provider roles). The final focus group consisted of ‘standard’ EMS providers. The research team were conscious that the phenomenon of interest was potentially controversial for clinicians to discuss candidly. The testing and refinement phase of the study allowed the methodology to be adjusted as the study progressed so that the final focus group with ‘standard’ EMS providers, the group of most interest, was completed successfully.

Four case vignettes (appendices one to four) were designed, reviewed and refined by the study team and discussed in the focus groups. The vignettes described the OHCA call information available initially, with further information given to the EMS providers over time to simulate the sequence of events and information that usually becomes available before and during an OHCA. A “sense checker” (Additional file 1) was developed to ensure that EMS providers would take a consistent approach to a routine OHCA scenario. The remaining vignettes described a palliative care scenario (Additional file 2), a paediatric traumatic OHCA (Additional file 3) and an incident where following 20 min of advanced life support the patient was in an organised cardiac rhythm but with no palpable pulse (pulseless electrical activity: PEA) (Additional file 4). The vignettes were deliberately vague to elicit the meaning participants ascribe to specific contexts that influence the decision-making process in OHCA resuscitation.

The four focus groups took place between February 2015 and July 2015. One researcher (KK) conducted the focus groups. Focus group discussions lasted approximately 1 h and were audio recorded and transcribed in full by an independent transcriber. Thematic analysis was carried out by JBr and checked with KK.

A well-established, iterative process of thematic analysis was used to analyse verbatim transcripts [14]. This included:

1. Familiarisation with the data: reading and re-reading transcripts
2. Generating initial codes: noting codes of interesting and pertinent ideas
3. Searching for themes: systematically organising these recurrent ideas with extracts of text
4. Reviewing themes: checking themes are meaningful and relate to the text
5. Defining themes: summarising the narrative with clear descriptions
6. Producing the report: using extracts of data to exemplify the themes

The data were scrutinised for similarity as well as conflicting ideas and concepts. The majority of themes were found in each group discussion and thus were consistent over all four groups. Data saturation was considered achieved, since no new concepts arose in the final group.

## Results

The demographic characteristics of participants are shown in Table 1. Data was collected to demonstrate the range of participants.

**Table 1:** Demographic characteristics of focus group participants

Age	Interquartile Range: 20.5 years Median age: 40 years
Gender	Female: 6 Male: 10
Parental status	Parental Responsibilities: 9 No Parental Responsibilities: 7
Educational route to paramedic registration	IHCD_*_ only: 4 IHCD plus higher education: 6 BSc_*_:3 FdSc_*_:1 DipHE_*_:2
Length of service	Interquartile Range: 13.5 years Median length of service: 15 years

*IHCD Institute of Healthcare Development, *BSc* Bachelor of Science, *FdSc* Foundation of Science, *DipHE* Diploma in Higher Education

EMS provider decision-making in OHCA is influenced by guidelines, protocol and policy. Additional influential factors on decision-making are well known to practitioners and other health professionals. They are less well understood in the literature, and not formalised in training, instruction and theory. Our findings are illustrated in figures one and two and described below. In addition extracts from the focus groups are used as examples of influential factors.

**Fig. 1:**
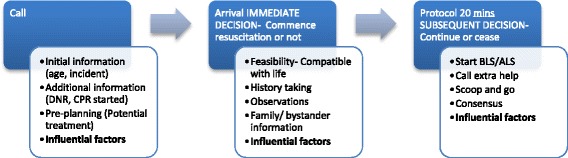
The stages of decision-making. The stages of decision making for EMS providers before arrival and continuing on scene

Figure 1 illustrates the stages of decision making for EMS providers before arrival and continuing on scene.

Stage 1. The initial call will usually include the type of incident and age and gender of the person as well as the location. This call gives information upon which the EMS provider and their colleagues will be starting to make decisions.

Stage 2. On arrival at scene the EMS provider will use their clinical reasoning to make a judgement about the feasibility of resuscitation as well as asking for more information from the people present; bystanders and/or family.

Stage 3. Assuming there is a chance of survival, or no obvious reason not to begin intervention, EMS providers will commence resuscitation.

**Fig. 2:**
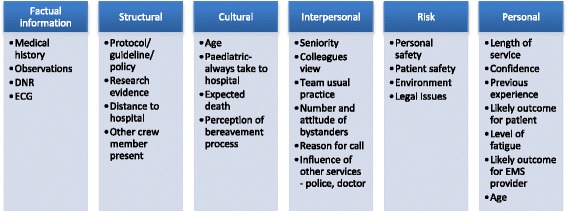
The influencing factors on EMS decision-making. The six domains of influencing factors in EMS provider decision-making

These stages appear logical and linear and, for those unfamiliar with pre-hospital care, it may be difficult to see how decision-making might vary from case to case. Nevertheless, influential factors present at each stage can lead to different decisions and variability in practice. The factors fall into six domains, shown in Fig. 2 and described in more detail below.

### Factual information

EMS providers use factual, seemingly definitive, information to make decisions about resuscitation. A primary source of information is the electrocardiogram (ECG) trace.

EMS providers are also aware that co-morbidities make the patient less likely to survive a resuscitation attempt. Having an advance directive with instructions not to resuscitate (DNACPR) gives EMS providers a clear understanding of the person’s wishes and whether to continue or not. However this can be difficult to adhere to when family members are distressed and desperately asking for their loved one to be saved:

*“M: …there’s a DNR in place, they’re dying … but there’s 3 daughters, a husband, 4 nieces, all saying ‘Don’t let him die, don’t let him die.’ So that’s quite a lot of pressure to ignore, isn’t it…If you’ve got the DNR…In your hand, then I would argue it. But it’s difficult.”*

*“F: You’d have a duty to argue [the] patient’s wishes.” (FG4)*

The vignette presented to the groups in this case specified that a DNACPR was believed to be in place, but could not be found. This led to considerable discussion between participants, since without it some felt they might face criticism or disciplinary procedures if they did not attempt resuscitation where no formal paperwork had been located.

### Structural factors

Protocols, guidance and policy used by ambulance services are structural factors providing a shared understanding of what is expected. Participants referred regularly to the minimum 20 min they would attempt resuscitation before concluding the attempt was futile. Several suggested that they often overran the time period to be sure they were giving the person the best chance of survival or the ‘benefit of the doubt’. Another described a holistic approach to decision-making, which supplements the underlying protocol:

*“M: … I’m thinking, OK, what caused the arrest? How can we treat that? What’s going to happen (when) they get to hospital? What’s their expected outcomes, what’s their current life situation? and thinking a lot more about the actual patient as an individual rather than just a protocol-driven management…” (FG2)*

One participant suggested his practice was also guided by research:

*“M: So, for me, I’m quite happy to step outside the guidelines as long as I’ve got confidence that the research behind me, I suppose….” (FG1)*

Distance to hospital was a factor in decision-making. This was related to the time taken to transport the person to hospital, the likelihood of survival, the viability of on-board resuscitation versus stabilisation of the person in situ and having help to carry out the resuscitation attempt.

*“F: Yes, if you’ve got long distance [you think] is there any way you can….even if it’s not a major hospital … can you get someone from somewhere? Can you get an extra pair of hands from somewhere?”…. (FG1)*

Because the ambulance service used in this study has a widely variable geographical area the participants suggested that their response in the city would be different to their response in rural areas.

*“F: I work rurally and I think the influence on my decision-making would be…it’s 20 miles to get to hospital… my decisions are made much more quickly… are we going to stay… are we going to go. It does affect it”. (FG3)*

### Cultural factors

The age of the person in cardiac arrest has an effect on EMS provider behaviour. In all focus groups participants said that when dealing with children they would always attempt resuscitation. This was the case even if there was little chance of survival, and appears to reflect a belief that the death of a child is unjust and untimely. Participants felt they would remove the child from the scene, taking them to hospital to give the child the best possible chance, or allow the parents to be supported by the hospital team if resuscitation proved unsuccessful.

*“F: If it’s likely to be a bad outcome you want the parent. The parent needs to be there for practical reasons at the hospital anyway, but the parent needs to be there if this is the end of their child’s life then no matter what happens… You’re always going to resus a child all the way into hospital …………. You are not going to do anything else. You need the parents to know even if we think the outcome might be very negative you want the parents to know that everything possible is being done.” (FG1)*

For adults who had not yet reached usual life expectancy, even if death was expected, discussion of age became a more contentious issue. For instance in one vignette, a young man is receiving palliative care but his advance directive cannot be found:

*“F: He’d be considered very young so I think there’s a feeling maybe that we should do things for someone who is much younger. I think the decision would be different if it was a 90-year old. So I think that age might influence a little bit even though it shouldn’t.” (FG3)*

This kind of decision was balanced between empathy for the person’s wishes at the end of their life and the perceived risk of litigation and disciplinary procedures:

*“M: it’s easier for them to think that the Trust can beat me with a stick but all I did was start CPR whereas .. they’ll think… Well, they could sack me for not starting potentially saving that patient’s life. There is that perception in place.” (FG3)*

### Interpersonal factors

It is common practice to secure consensus to cease resuscitation if several EMS staff are present. This works well if all are in agreement. However, there are situations where interpersonal factors complicate the decision. There was a generally agreed position not to over-rule someone in doubt. However one participant noted that this preference may be misplaced:

*“M: …because you have to respect your colleagues’ wishes, don’t you almost it’s a bit of a shame [they are] almost stronger than the wishes of the person who is laid on the bed which is a bit ironic, isn’t it, because you are there for them and not for you.” (FG4)*

There are situations where it is not clear what the best course of action is. The participants suggested that in these cases a doctor’s decision becomes necessary:

*“F: …because when you call a doctor to a cardiac arrest you call them for one of two reasons; either to help get them back or because of circumstances that you can’t call it… We couldn’t technically stop the resus until a doctor gave us permission.” (FG1)*

One particular respondent was clear that this kind of escalation protected the EMS provider. This was influenced by an experience of having to defend decisions made in formal court proceedings.

For some of the focus group participants there was a notable difference between their responses relating to the team they worked in and the type of work they encountered on a daily basis. The ‘standard EMS providers’ were more likely to see the ‘grey areas’ in their decision-making, while the specialist teams were more likely to be ‘protocol-adherent’, and be very clear about the response they were expected to make.

The presence of onlookers and particularly parents, in the case of children, was a compelling reason to commence resuscitation and remove the person from the scene.

*“M: So the crews, the police, the grandmother, the 50 people that were there, were all expecting this child to go in the back of an ambulance somewhere, and you just know immediately if you think ‘just call it’ it would just bring a whole heap of trouble. I think most of the doctors would have done the same, exactly the same, because you read the situation, don’t you?” (FG2)*

Other people at the scene can also be influential, such as police officers. One participant described a case of suspected suicide by hanging. The police officers had not attempted to resuscitate the patient thinking it was too late and the EMS providers found it difficult to dispute this.

### Risk factors

The participants described several occasions when they might be influenced by perceived risk to themselves or others. For instance one vignette described a person who was obese. The participants deliberated over this when making the decision about extrication. Removing a person who is heavy could delay the process and thus the chance of success, but also put the EMS providers at risk of physical injury.

### Personal factors

Some of the participants talked about their length of service as a measure of experience; this affected their confidence in their own decision-making:

*“M: … all of this, I think, is based on past experience of the clinician. So if you go to that job and you get it wrong… whichever decision you make you get it wrong… you will never do that again.” (FG3)*

This illustrates how EMS providers come to each scene with pre-existing influences; whether these are factual or perceptual, they continue to prime the EMS providers’ expectations of success. This is mediated by the length of service, type of training and whether they feel their decisions will be supported by their employer:

*“M: So this is clinician confidence now. it would be really interesting to do this with a bunch of graduates who are in their first 6 months post graduation because we do have a duty to not provide an unethical service so if that is a child that is not going to survive we can’t do it, is my mind, but that’s clinician confidence. I think it depends on people’s past experiences and depends on whether people trust and support them to make these decisions because arguably nowhere in the Trust guidelines does it say that that we’ve got a duty to withhold unethical services…” (FG3)*

Participants described how EMS providers can suffer anxiety after the event. This might be due to concern as to whether they acted in the best interests of the patient or concern about the possibility of litigation and disciplinary action. One participant described the difficulty in deciding to resuscitate when there is no formal evidence of a DNR, even when the family say it exists:

*“M: I don’t see the point in half-doing … you either commit to the decision that patient has made… or if there’s enough doubt then I’d say I am ever so sorry but we’re going to have to do resus… it’s the law…”*

*“M: And you wouldn’t have the 2 or 3-week period afterwards where you were worrying about [it]” (FG4)*

The interviewer raised the issue of EMS provider fatigue and calls that came at the end of a long shift. Participants suggested some would accelerate the decision to extract the person and take them to hospital, while others would try even harder at the scene so they couldn’t be accused of doing less at the end of a shift, nor doubt their own efforts.

*“M: Time of day would have a bearing on the time shift. If it’s 20 minutes before the end of my shift this is going… because if I stay here I could be here for another hour so if it’s 20 minutes to the end of shift I might as well take it into A&E my job’s done and I can finish on time. That’s me being cynical”.*

*“M: See my take on towards the end of shift is staff become over cautious at the end of shift and do more than they need to so they can’t be criticized for. Well it was the end of your shift”.*

*“F: And they don’t want to go home thinking about it as well” (FG3)*

## Discussion

This study used discussion groups with EMS providers to understand contemporary practices and beliefs surrounding decision-making during resuscitation. The analysis indicates three stages of decision-making and six domains of influencing factors.

These accounts go beyond the pragmatic or logistical factors noted by Hick et al. [7], and are more in keeping with the results reported by Naess and colleagues, [6] who considered and integrated a range of prognostic and ethical criteria used in decision-making from the perspective of the patient, bystander, paramedic and wider community. Grudzen et al. [8] also identified provider and cardiac arrest characteristics that are important in decision-making, including the personal and interpersonal beliefs and dynamics of the paramedic, patient and onlooker.

**Fig. 3:**
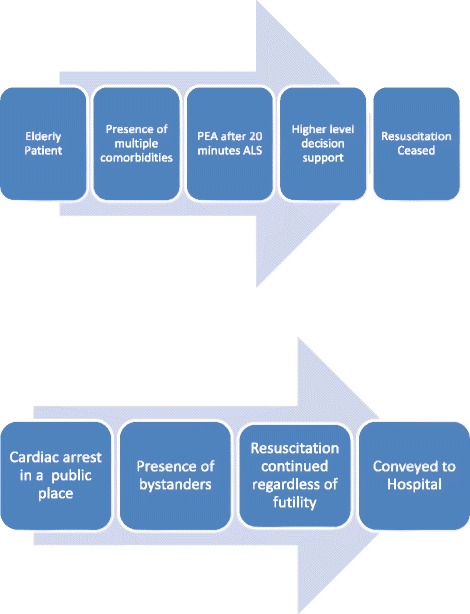
Examples of EMS provider resuscitation decision-making based on research findings. Figure 3 shows two examples illustrating the process of EMS decision-making when commencing and ceasing OHCA

We have described and illustrated the complex and often conflicting information and perspectives available to an EMS provider when they are making decisions about resuscitation. Two examples illustrating the process of decision-making are shown in Fig. 3. Many of these decisions are not clear-cut and amenable to a protocol-driven approach, since variations in context and circumstances are substantial. This does not mean that protocols are irrelevant, since they appear to be valued as a basis for decision-making, especially when dealing with factual information such as ECG interpretation. It is, however, the additional circumstantial, individual and interpersonal characteristics that mediate the decision-making process and the practical application of formal guidelines.

The participant views examined here give rational accounts of cases encountered, and do not necessarily divulge the less rational, value or assumption based influences on decision-making. For example, when considering obesity, rational arguments were advanced regarding risk to EMS staff and the patient. Age-related influences are more obviously socially sanctioned, and seemed easier to consider as a factor in decision-making.

Although the age, status and training route of each participant was collected, to characterise our EMS provider sample, we have made no association between these factors and the views expressed. However it appeared that specialist teams had more certainty in their approach to resuscitation. This may be attributable to their greater experience of cardiac arrest and its management.

Our study has some important limitations. It was completed in one ambulance service in England, and thus generalisation nationally and internationally may be limited as EMS systems have different processes and systems in place. A criticism of the focus groups and vignette methodology is that the responses given may have been influenced by other participants in the group. In addition, although the vignettes were based on ‘real life’ examples it is impossible to emulate the unpredictable and dynamic scene of an OHCA and to bring this context into the focus groups. In the focus groups the participants had more time in a calm environment to consider what their actions may be. Progressing further research in this area requires careful consideration of how to overcome this challenge.

These research findings provide the background to refine and progress this work further with the aim of allowing a comprehensive understanding of the influences on EMS resuscitation providers’ decision-making when commencing and ceasing OHCA resuscitation. Our study has indicated variability between EMS providers in their decisions to commence and cease resuscitation attempts. A clearer understanding of the influential factors that contribute to EMS decision-making in this situation has the potential to allow these factors to be recognised in clinical guidelines, and included in both the education of EMS providers and the mechanisms used for clinical decision support. A more uniform approach to decision-making in this area has the potential to improve patient outcomes and resource use within EMS systems.

## Conclusions

There are three distinct stages in the EMS provider decision-making process when deciding whether to commence or cease resuscitation attempts in OHCA. These stages are: the call; arrival on scene; the protocol. Influential factors present at each of the three stages can lead to different decisions and variability in practice. These are: factual information available to the EMS provider; structural factors such as protocol, guidance and research; cultural beliefs and values; interpersonal factors; risk factors; personal values and beliefs. Improved understanding of the circumstantial, individual and interpersonal factors that mediate decision-making in practice can inform the development of more effective clinical guidelines, enhanced education and clinical decision support. Improved consistency and EMS clinician confidence has the potential for improved patient outcomes following OHCA.

## Additional files

Additional file 1: Appendix one – Case Vignette – Sense checker. (DOCX 15 kb)

Additional file 2: Appendix two – Case Vignette - Palliative. (DOCX 15 kb)

Additional file 3: Appendix three – Case Vignette – Paediatric trauma. (DOCX 18 kb)

Additional file 4: Appendic four – Case Vignette – Carry on or call? (DOCX 16 kb)
