# Supplementary material for: Emergency medical service provider decision-making in out of hospital cardiac arrest: an exploratory study
Source: BMC Emerg Med. 2017 Jul 25;17:24. doi: 10.1186/s12873-017-0136-3 (PMC5526270; doi:10.1186/s12873-017-0136-3)
Supplement: Supplementary file 1 — Appendix one – Case Vignette – Sense checker. (DOCX 15 kb) [file 12873_2017_136_MOESM1_ESM.docx]

## Additional file 1

## Appendix One

Case Vignette: Sense checker

Initial Detail: 75 year old male. Witnessed collapse. Query cardiac arrest. Wife doing CPR. Estimated time to arrival 9 minutes

**Scenario**

Once crew has arrived the patient stays in VF for 5 minutes or (2 shocks).

The patient then remains in asystole for the remainder of ALS.

- He has not vomited
- He is easily intubated
- He is easy to cannulate
- The patient’s wife is distressed

**Possible Questions From Paramedics?**

Did you see what happened, what exactly happened?

He came in from the garden complaining of chest pain and collapsed in the kitchen. He made some funny noises, seemed to have a bit of a fit and was unconscious. His wife called 999 straight away and followed the dispatcher’s instructions to do CPR and carried on until the ambulance arrived.

Has your husband been well recently?

Yes, he’s been a bit breathless, but fine.

Has he seen a doctor recently?

No, he hates going to the doctor

What is his medical history?

Arthritis, angina, high blood pressure.

Do you have a list of his medications?

GTN, Simvastatin, Paracetamol, Amlodopine, Bendroflumethiazide

Did you start doing CPR straight away?

Yes, as soon as the ambulance person told me what to do.

Do you know if your husband would have wanted to be resuscitated?

We never discussed it.
