# Supplementary material for: Emergency medical service provider decision-making in out of hospital cardiac arrest: an exploratory study
Source: BMC Emerg Med. 2017 Jul 25;17:24. doi: 10.1186/s12873-017-0136-3 (PMC5526270; doi:10.1186/s12873-017-0136-3)
Supplement: Supplementary file 2 — Appendix two – Case Vignette - Palliative. (DOCX 15 kb) [file 12873_2017_136_MOESM2_ESM.docx]

## Additional file 2

## Appendix Two

Case Vignette: Palliative Scenario

Initial Detail: Incident time 0436. 39 year old male query cardiac arrest. The patient has cancer, he has had problems breathing and become distressed in the night. Now query deceased.

**Scenario**

There has been no CPR before ambulance arrival.

Initial rhythm is VF.

PEA after one shock.

Asystole after 15 minutes

- He has not vomited
- Air entry is difficult. Intubate on 2^nd^ attempt
- Unable to get IV access
- Can get IO access if attempted
- Parents are very distressed. They don’t want the patient to be resuscitated. They called because they didn’t know what else to do.
- Unable to produce DNR

**Possible Questions From Paramedics?**

Did you see what happened, what exactly happened?

He has been very unsettled all night. Parents are waiting for an OOH GP visit to see if anything can be done to settle the patient. He was finding it hard to breathe and then his breathing became much more shallow. He then seemed to stop breathing. The parents didn’t know what to do so called 999.

What is his medical history?

Bowel cancer, but it has spread. He has had cancer for 2 years. He was given 18 months to live 2 years ago.

What medical treatment is your son receiving?

He is under the district nursing team, they visit every day.

What medications does your son have?

There’s a syringe driver in place, morphine, hyoscine

Did you start doing CPR straight away?

No we haven’t as he didn’t want to be resuscitated.

Do you have a copy of the DNR

We do, but we can’t find it.
