# Supplementary material for: Emergency medical service provider decision-making in out of hospital cardiac arrest: an exploratory study
Source: BMC Emerg Med. 2017 Jul 25;17:24. doi: 10.1186/s12873-017-0136-3 (PMC5526270; doi:10.1186/s12873-017-0136-3)
Supplement: Supplementary file 3 — Appendix three – Case Vignette – Paediatric trauma. (DOCX 18 kb) [file 12873_2017_136_MOESM3_ESM.docx]

## Additional file 3

## Appendix Three

Case Vignette: Paediatric Trauma Scenario

Initial Detail: You are called as a crew to an RTC, when the details update, there is one patient involved, a 3 year old male. You receive an update and radio alert to inform you the patient is ? cardiac arrest and the patient’s father is doing CPR.

- You are the first crew on scene at 0907 and the call origin was 0855.
- The patient is confirmed to be in cardiac arrest and the presenting rhythm is asystole.
- The patient has a severe head injury
- Continues in asystole
- IO on 2^nd^ attempt
- Airway managed initially by OP airway and then LMA
- Air Crew unavailable
- Lots of bystanders
- Patient’s hysterical Mother arrives
- 2^nd^ crew and duty office arrive simultaneously at 0918

**Possible Questions From Paramedics?**

Did you see what happened, what exactly happened?

An hysterical father tells his son was running and tripped and fell into the path of a car

What is his medical history?

He is well

Was CPR started straight away?

Yes my husband is a first aider

.
