# Supplementary material for: Emergency medical service provider decision-making in out of hospital cardiac arrest: an exploratory study
Source: BMC Emerg Med. 2017 Jul 25;17:24. doi: 10.1186/s12873-017-0136-3 (PMC5526270; doi:10.1186/s12873-017-0136-3)
Supplement: Supplementary file 4 — Appendic four – Case Vignette – Carry on or call? (DOCX 16 kb) [file 12873_2017_136_MOESM4_ESM.docx]

## Additional file 4

## Appendix Four

Case Vignette: Carry on or call?

Initial Detail: Incident time 2006. 83 year old female collapsed upstairs trapped behind the bathroom door. The caller is unsure if she is breathing. The patient’s husband is on scene and is very distressed.

- On arrival (9 minutes from call origin (20:15) you are shown where the patient is by the very distressed spouse of the patient. The patient is collapsed behind the toilet door, but you are able to shift the patient by forcing the door with your crew mate’s help.
- Once access is gained the patient is confirmed in cardiac arrest and dragged onto the landing. The crew note the patient is obese and possibly 20 stone.
- 2^nd^ crew is en route.
- 1^st^ rhythm analysis at 20:17 - Patient is in PEA.
- Difficulty in obtaining IV access – gained on 3^rd^ attempt (20:21)
- 20:22 Rhythm and pulse check. PEA.
- Unable to intubate. Igel inserted successfully at 20:26 and airway is secured.
- 2^nd^ adrenaline given at 20:27
- 3^rd^ rhythm and pulse check at 20:28 – asystole.
- ALS continues. 3^rd^ adrenaline.
- 4^th^ rhythm and pulse check at 20:31 patient is in PEA, pupils fixed and dilated.
- ALS continues.
- Patient’s daughter arrives vey distressed.
- A 2^nd^ crew arrives and a handover is given.
- 5^th^ rhythm and pulse check 20:33 – PEA – ALS continues.
- 6^th^ rhythm and pulse check at 20:36 – PEA.

**Possible Questions From Paramedics?**

Did you see what happened, what exactly happened?

She was in the bathroom, I heard a bang and when I got up the stairs I couldn’t open the door. She has been in there for 10 minutes.

What is her medical history?

The patient has been complaining of back pain recently, diabetes type 1, hypertension, angina, sleep apnoea and poor mobility. She has been feeling unwell recently and is prone to falls

What medical treatment is your wife receiving?

She sees the doctor regularly and has medications

What medications does your wife have?

He can’t remember and goes off to find them. He is very distressed and begging the crew to save her.

Has your wife ever discussed whether she wants to be resuscitated

No, never.
